# The role of exosomal non-coding RNAs in cancer metastasis

**DOI:** 10.18632/oncotarget.23552

**Published:** 2017-12-21

**Authors:** Jin-Peng Wang, Yan-Yan Tang, Chun-Mei Fan, Can Guo, Yan-Hong Zhou, Zheng Li, Xiao-Ling Li, Yong Li, Gui-Yuan Li, Wei Xiong, Zhao-Yang Zeng, Fang Xiong

**Affiliations:** ^1^ The Key Laboratory of Carcinogenesis of the Chinese Ministry of Health, Xiangya Hospital, Central South University, Changsha, Hunan, China; ^2^ The Key Laboratory of Carcinogenesis and Cancer Invasion of the Chinese Ministry of Education, Cancer Research Institute, Central South University, Changsha, Hunan, China; ^3^ Hunan Key Laboratory of Nonresolving Inflammation and Cancer, Disease Genome Research Center, The Third Xiangya Hospital, Central South University, Changsha, Hunan, China; ^4^ Department of Cancer Biology, Lerner Research Institute, Cleveland Clinic, Cleveland, OH, USA

**Keywords:** exosome, non-coding RNA, tumor microenvironment, tumor metastasis

## Abstract

An increasing number of studies has confirmed that many cells can secrete vesicles or exosomes in eukaryotes, which contain important nucleic acids, proteins and lipids and play important roles in cell communication and tumor metastasis. This paper summarizes the comprehensive function of exosomal non-coding RNAs. Although some studies have shown that exosomes mediate tumor signal transduction, the functional mechanism of the tumor metastasis remains to be elucidated. In this paper, we reviewed the role of exosomal non-coding RNAs in mediating cancer metastasis in the tumor microenvironment to provide new ideas for the study of tumor pathophysiology.

## INTRODUCTION

Tumor metastasis is the main cause of cancer death [1–6]. Metastasis is a multiple-level process that includes tumor cell transit through the basement membrane into the vasculature, survival in the circulation system, gradual outward invasion, and finally colonization to distant organs, dissemination of cancer cells and adaptation to the microenvironment to promote cancer development [7–12]. In the process of tumor metastasis in the tumor microenvironment, studies have confirmed that cancer cells communicate with each other and that the surrounding stromal cells could lead to the occurrence and development of tumor metastasis [13–20]. Exosomes play important roles in the tumor microenvironment and the mechanism of malignant tumor metastasis.

**Figure 1:**
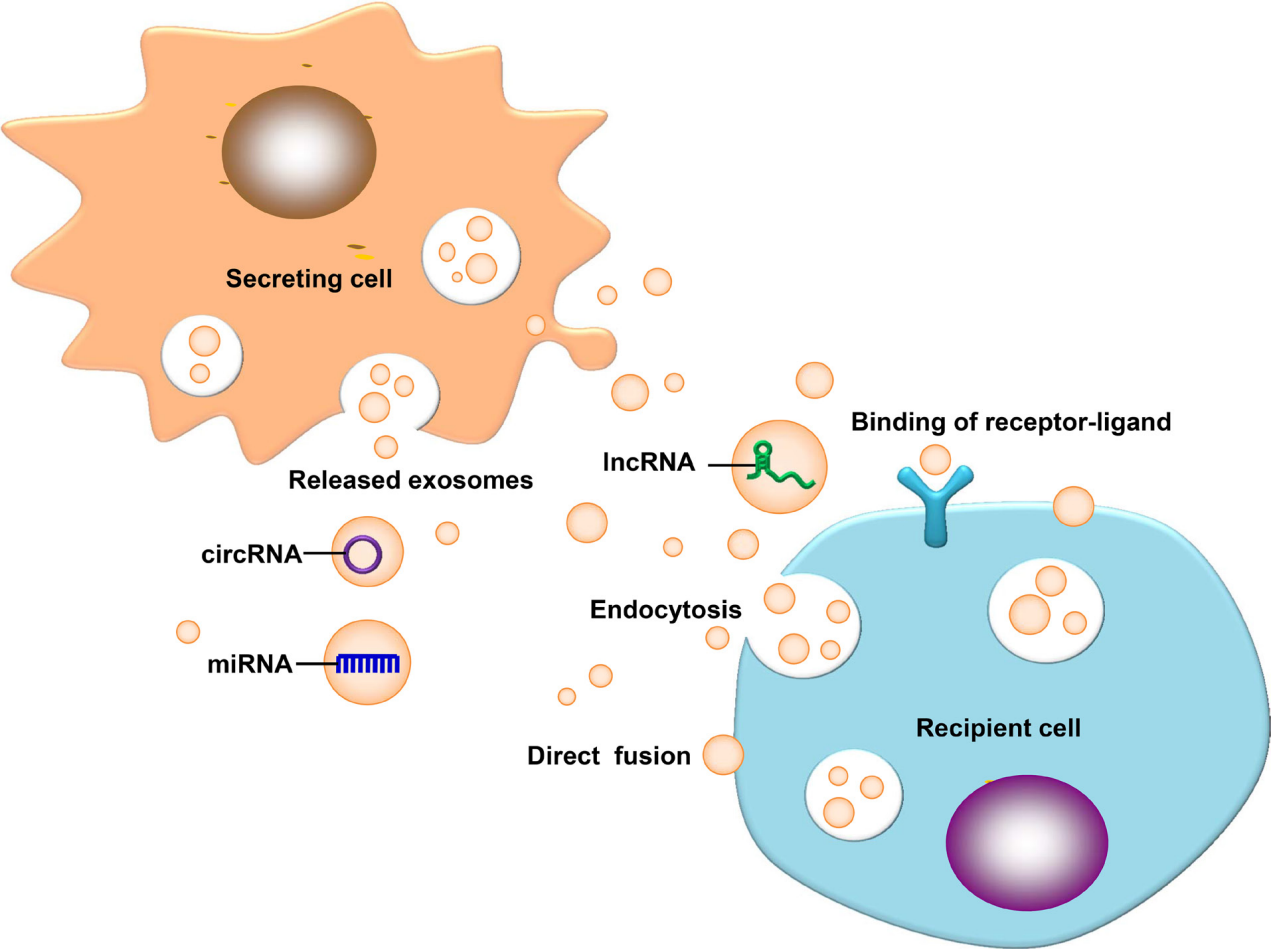
Exosomal ncRNAs ingested by a receptor cell Exosomal ncRNAs ingested by a receptor cell via direct fusion, endocytosis and binding of receptor ligands.

Exosomes consist of a phospholipid bilayer, which is composed mainly of proteins, lipids, carbohydrates and nucleic acids [21–23]. Studies on exosomal non-coding RNAs (ncRNAs) have raised in recent years, such as microRNAs (miRNAs), long non-coding RNAs (lncRNAs), and circular RNAs (circRNAs). Exosomes can carry these functional biomolecules to receptor cells to promote non adjacent intercellular communication [24, 25]. The main sources of exosomes in lysosome particles include invagination and the formation of multi-vesicular bodies, multi-vesicular bodies and serosa release exosomes: receptor cells can ingest exosomes via direct fusion, endocytosis or fusion with exosome surface markers [26]. Exosomes can transmit information to the extracellular matrix to regulate the receptor cells [27–29]. The function of exosomes depends on the cell type [29], which may be involved in the immune response, antigen presentation, cell migration, cell differentiation, tumor invasion and other aspects [30]. The above concepts are illustrated in Figure 1.

Exosomes promote the growth of tumor cells in recipient cells by promoting angiogenesis, invasion, proliferation and diffusion [31, 32]. Exosomes target to stromal cells, immune cells and vascular cells in the tumor microenvironment to promote the occurrence and development of cancer [33]. Studies have shown that exosomes can directly regulate the invasion and migration of tumor cells and can promote the directional movement of cells via the activity of biological components of the extracellular matrix (ECM) [34, 35]. Tumor exosomes can promote the growth and spread of tumor cells by affecting the permeability of blood vessels or anchoring distant metastasis sites to change the physiological functions of adjacent or distant cells [36–40]. In this review, we focus on the study of the role of exosomal non-coding RNAs in the development of cancer, which are important molecules in cancer metastasis.

## THE ROLE OF EXOSOMAL NON-CODING RNAS IN TUMOR METASTASIS

### Exosomal miRNAs in tumor metastasis

MicroRNAs (miRNAs) are approximately 22-nucleotide-long small molecules of RNA. miRNAs bind to the target mRNA 3 'UTR to prevent the translation of the target mRNA or promote the degradation of target mRNA at the post transcriptional level, to negatively regulate the target genes, and thus to inhibit or promote tumor metastasis [41–43]. Recently, exosomal miRNAs have been found to play key roles in physiological and pathological processes in eukaryotes [44–47]. The tumor-derived miRNAs and stromal cells of the microenvironment are interrelated with each other, which regulate the tumor progression, angiogenesis, invasion and metastasis and can escape from immune surveillance [48]. The role of exosomal miRNAs in tumor metastasis is summarized in Table 1.

**Table 1:** The biological functions of exosomal miRNAs in tumor metastasis

Exosomal miRNA	Role in tumor metastasis	Tumor type	Reference
*miR-23a*	Induce EMT by targeting TGF- β Directly target PHD1/PHD2 and ZO1 to promote lung cancer cell permeability, angiogenesis and migration across endothelial cells	Lung canner Lung cancer	[54] [76]
*miR-19a*	Promote brain metastasis by inducing PTEN loss	Breast cancer	[8]
*miR-122*	Regulate niche cells, glycolytic enzymes, pyruvate kinase, inhibit glycolysis, and promote tumor metastasis	Breast cancer	[65]
*miR-24-3p*	Targeting FGF11 to inhibit the function of T cells	Nasopharyngeal carcinoma	[66]
*miR-134*	Inhibit STAT5B, Hsp90 and Bcl2, thereby inhibiting tumor proliferation, invasion and metastasis while promoting cisplatin-induced apoptosis	Breast cancer	[71]
*miR-939*	Inhibit VE-cadherin and destroy endothelial barrier function	Breast cancer	[72]
*miR-1246*	Regulate cell movement and invasion by regulating DENND2D of oral squamous cell carcinoma	Oral squamous cell carcinoma	[68]
*miR-105*	Targeting ZO-1 damages vascular endothelial cells and promotes metastasis	Breast cancer	[67]
*miR-200*	Directly target the mRNA transcriptional repressor ZEB1, E-cadherin, ZEB2, up-regulate the expression of E-cadherin, and inhibit motility in cancer	Ovarian cancer	[74]
	It is absorbed by recipient cells so they can develop metastasis	Breast cancer	[70]
*miR-193a*	Interaction with the MVP, which can block the release of miR-193a after exocytosis, can cause the accumulation of miR-193a in donor cells and inhibit tumor development	Colon cancer	[28]
*miR-142-3P*	It is selectively sorted out to promote malignant phenotypes both inside and outside the cell	Oral carcinoma	[79]
*miR-320a*	MiR-320a-PBX3 inhibits the activation MAPK pathway and the occurrence of EMT, and it down-regulates the expression of CDK2 and MMP2, thereby inhibiting tumor development	Liver cancer	[80]
*miR-21*	Exosomes transport the stromal cell derived miR-21 to ovarian cancer cell. MiR-21 targeting APAF1 induces resistance to paclitaxel in ovarian cancer cells	Ovarian cancer	[63]
	Down-regulation of PTEN to activate the PIK3/AKT signaling pathway and promote the cisplatin resistance of gastric cancer cells	Gastric cancer	[120]
*miR-155*	Exosomes transport miR-155 to other PDAC cells, effectively attenuating drug resistance	Pancreatic ductal adenocarcinoma	[73]
*miR-214*	Down-regulation of the expression of PTEN protein results in the secretion of large amounts of IL-10 by Tregs, which results in the immune escape of the host and tumor growth	Lung cancer	[117]
*miR-221*	Exosomes transfer miR-221 to tumor cells and can bind to hormone therapy targets, resulting in tumor HTR	Breast cancer	[122]
*miR-138*	The expression of miR-138 can increase angiogenesis. CTU1, KIAA1274 and RAX regulate KNG1 by miR-138	Glioma	[77]

Epithelial-mesenchymal transition (EMT) refers to the loss of the characteristics of epithelial cells and gaining the process of interstitial cell phenotype. This process includes changes in the cell’s morphology and phenotype, with invasion and distant metastasis of tumor cells as the initial step [49–51]. Scholars confirmed that stromal cell-derived exosomes transport miRNAs to tumor cells, leading to EMT changes in the morphology and biochemistry of tumor cells. Intercellular communication also affects the occurrence and development of the EMT process. Research has shown that exosomes contain special proteins and miRNAs that could induce phenotypic changes [52]. Recently, researchers have confirmed that exosomes contain *miR-23a*, which can influence the occurrence and development of EMT by regulating TGF-β. Exosomes induce the activation of TCF/LEF and the Wnt pathway in lung cancer cells, which affect the occurrence and development of tumors [53–55]. Exosomal miRNAs can be transferred to the target cells, where they directly modify their target mRNAs [56–60].

The development of tumor metastasis requires the tumor cells to adapt to the new metastasis site. However, for when and how the tumor cells can develop metastasis, the underlying mechanisms remain unclear. Recently, researchers have shown that when a primary tumor reaches to the brain, tumor cells that normally express *PTEN* lose its expression. When *PTEN-*deficient metastatic brain cancer cells leave the brain, the expression of *PTEN* will return to normal levels, and the reversal of *PTEN* deletion occurs via astrocyte-derived exosomal *miR-19a* in the brain and spinal cord. The *miR-19a* targets *PTEN* to complete the biological behavior [8]. The loss of *PTEN* in brain tumor cells could increase the secretion of cytokine CCL2, which can restore microglia to metastatic tumors to promote the growth of tumor cells and can protect cancer cells from death. This study provides a new hope for the treatment of cancer metastasis [61–64].

Glycolysis and carbohydrate uptake are aberrantly active during carcinogenesis. Recent studies have shown that exosomal *miR-122* can be found in breast cancer cells, which can inhibit the proliferation of primary tumor and increase the effectiveness of nutrients of the precancerous metastasis niche, thereby inducing cancer metastasis. *miR-122* inhibits carbohydrate uptake by niche cells by inhibiting glycolytic enzymes and pyruvate kinase. The inhibition of *miR-122* can reduce the incidence of brain and lung metastases *in vivo* [65]. By changing the efficiency of glucose utilization by the susceptible anterior metastatic niche cells, exosomal *miR-122* can be adapted to the system’s energy metabolism program to promote cancer progression.

DNA methylation, histone modification and miRNAs play important roles in the process of tumor progression by regulating the microenvironment and immune surveillance. Exosomal miRNAs can not only be used as potential markers of malignant tumors, but they are also involved in the internal communication between cancer cells and the microenvironment. The expression of *miR-24-3P* was significantly higher in the plasma of nasopharyngeal carcinoma patients. The *miR-24-3P* inhibited the proliferation of T cells and the differentiation of Th1 and Th17 and induced the regulation of T cells. *MiR-24-3P* regulated the proliferation and differentiation of T cells, decreased the expression of P-ERK, P-STAT1 and P-STAT3, and increased the expression of P-ATAT5 in cells [66]. *FGF11* is a direct target of *miR-24-3P* in the regulation of proliferation and differentiation of T cells. Many studies have demonstrated that exosomal miRNA is involved in regulating the tumor microenvironment [67–71].

Exosomes can be involved in tumor resistance through intercellular communication by carrying biological information and drug factors. Studies have shown that the occurrence and development of breast cancer are closely related to *miR-134* and that the expression level of *miR-134* can reduce STAT5B, Hsp90, and Bcl-2 to reduce cell proliferation and promote apoptosis by cisplatin [71]. This study suggests that *miR-134* can be used as a diagnostic biomarker of breast cancer and may be a potential therapeutic target. Other studies have shown that exosomal *miR-939* is associated with poor breast cancer prognosis, inhibits VE-cadherin and destroys the barrier function of endothelial cells [72].

Gemcitabine (GEM) is a key drug used in pancreatic ductal adenocarcinoma (PDAC), but PDAC cells can produce chemical resistance after long-term administration. Because resistance is rapidly expanding into PDAC tissue, it is important to look for the mechanisms that produce resistance to chemotherapy. Researchers found that the long-term use of GEM increased the expression of *miR-155* in PDAC cells [73]. The increase in *miR-155* induced the secretion of exosomes via chemotherapy resistance, which promoted anti-apoptotic activity. Exosomes deliver *miR-155* to other PDAC cells. Targeting *miR-155* may thus effectively reduce drug resistance. This study found a new therapeutic target for GEM in PDAC as well as a new approach to address metastasis.

Studies have revealed breast cancer-derived exosomal *miR-200*, which can be taken by recipient cells to acquire metastatic ability [70]. Exosomal *miR-1246* can induce cell invasion and migration by regulating the DENN/MADD Domain containing 2D (DENND2D) in breast cancer cells [68]. Exosomal *miR-105* could destroy vascular endothelial cells to promote the occurrence and metastasis of ovarian cancer by targeting the tight junction protein ZO-1 [67]. Exosomal *miR-200* can directly target E-cadherin’s mRNA transcriptional repressors ZEB1 and ZEB2 to increase the expression of E-cadherin and inhibit the movement ability of tumors [74, 75]. Other studies have shown that exosomal *miR-23a* increased significantly in hypoxic lung cancer cells. *miR-23a* can promote angiogenesis by directly targeting prolyl hydroxylase1/2 (PHD1/PHD2), leading to the accumulation of hypoxia-inducible factor-1α (HIF-1α) in endothelial cells. At the same time, exosomal *miR-23a* inhibits the tight junction protein (ZO-1), thereby increasing the vascular permeability and migration ability. Hypoxic lung cancer cell-derived exosomal *miR-23a* can promote cancer progression by modulating the tumor vasculature [76].

Glioblastoma is a highly invasive malignant brain tumor that is highly vascularized and associated with high mortality. Kininogen-1 (KNG1) is associated with tumor suppression and antiangiogenic activity in glioblastoma. CTU1, KIAA1274 and RAX regulate KNG1 via *miR-138*. Following the knockdown of the expression of CTU1, KIAA1274 and RAX in U87 cells and immortalized human endothelial cells (iHEC), KNG1 expression is down-regulated, which results in the up-regulation of oncogenic EGFR signaling and induces angiogenesis in a mouse xenograft model. Knockdown of the expression of KNG1, CTU1, KIAA1274 or RAX can increase the expression of U87 cell-derived exosomal *miR-138* and enhance the angiogenesis of iHEC to promote cancer development [77].

Exosomes are new mediators of intercellular communication. In addition to the fact that the exosomes can affect the regulation of recipient cells, whether the release of exosomes affects donor cells remains uncertain. Recently, scholars have innovatively proposed that major vault protein (MVP)-mediated tumor suppressor miRNAs can be selectively separated to the body to promote the development of the tumor process [28, 78]. Many studies have confirmed that *miR-193a* can interact with the MVP and that the MVP promotes the release of *miR-193a* from the donor cells via the circulation of blood, consequently promoting tumor development. The amount of exosomal *miR-193a* released would decrease after knocking down the MVP, which would result in the accumulation of *miR-193a* in cells and decrease the development of tumor. *miR-193a* raises from the Ccnd2 and cMyc by targeting Caprin1, which retards the cell cycle G1 phase and inhibits cell proliferation. At the same time, they proposed that advanced patients had higher levels of exosomal *miR-193a*.

Through further investigation, we found that oral cancer cells can release *miR-142-3P* by selecting the extracellular vesicles, thus promoting the malignant phenotype both inside and outside the cells [79]. Inhibiting the exosomal protein Rab27A output, which can stop the release of exosomes, is thought to increase the expression levels of *miR-142-3p, miR-150-5p,* and *miR-223-3p*. Increasing *miR-142-3p-*targeted TGFBR1, which can downgrade the expression of TGFBR1 in donor cells, can reduce the formation of the malignant phenotype. The reverse validation by increasing the release of *miR-142-3p* in the donor cells, which can be ingested by receptors on endothelial cells, reduced the activity of TGFBR1, eventually enabling the development of tumor.

The loss of cancer-associated fibroblast-derived exosomal *miR-320a* can promote the proliferation and metastasis of hepatocellular carcinoma cells. *MiR-320a* can be combined with the downstream target genes PBX3 as a tumor suppressor miRNA to inhibit the liver cancer cell proliferation, migration and metastasis. *miR-320a-PBX3* inhibits the active pathway of the MAPK and EMT and reduces the expression of dependent cytokinase 2 (CDK2) and MMP2 to inhibit tumor development [80].

### Exosomal lncRNAs in tumor metastases

LncRNA are nucleotides longer than 200 nucleotides in length that do not encode proteins. The approaches to regulate genes of lncRNAs are varied. One of the most important ways is to directly target the transcription of target genes through base complementation and binding to target genes or by indirectly regulating target genes upstream or downstream of gene transcription [62, 81–88]. Although this process plays an important role in tumor development, the mechanisms underlying the relationship between lncRNAs and tumor metastasis need further study to identify more treatment approaches [17, 89–93]. The role of exosomal lncRNAs in tumor metastases is summarized in Table 2.

**Table 2:** The biological functions of exosomal lncRNAs in tumor metastasis

Exosomal lncRNA	Role in tumor metastasis	Tumor type	Reference
*HOTAIR,* *MALAT1,* *MEG3*	As a target for the early diagnosis of cervical cancer	Cervical cancer	[100]
*lncARSR*	The competitive combination of miR-34/miR-449 increase the expression levels of AXL and c-MET and promote the dissemination of sunitinib resistance through exosomes	Renal carcinoma	[60]
*H19*	Increasing the expression of VEGF and its receptor VEGF-R1, which may serve as a potential target for HCC	Liver cancer	[101]
*ZFAS1*	Promote the proliferation and migration of gastric cancer cells through the transmission of the exosome	Gastric cancer	[96]
*LINC00152*	The expression in exosome increased significantly in patients	Gastric cancer	[99]
*TUC339*	Regulating the microenvironment of hepatoma cells by intracellular transfer of the exosome, thereby regulating the growth and adhesion of tumor cells	Liver cancer	[95]

Recent studies have confirmed that lncRNA *HOX* transcript antisense RNA (*HOTAIR*) is associated with poor prognosis for many types of cancers. There are a variety of lncRNAs in the urine of bladder cancer patients, including *HOTAIR, HOX-AS-2, the MALAT1, SOX2, OCT4 HYMA1, LINC00477, LOC100506688,* and *OTX2-AS1.* Knockdown of *HOTAIR* in bladder cancer cell lines will reduce invasion and migration. More significantly, the loss of the *HOTAIR* will affect the changes of related EMT genes, e.g., *SNAI1, TWIST1, ZEB1, ZO1, MMP1, LAMB3,* and *LAMC2* [62]. The exosomal lncRNAs originating from urine can be used as new molecular markers and targets for molecular therapy, thus providing cancer treatments with more effective solutions.

CD90+ cancer cells are cancer stem cells that can promote cancer invasion and metastasis. The exosomes released by CD90+ cells (not parent liver cancer cells) are rich in lncRNA*H19*, which can regulate endothelial cells and promote angiogenesis and adhesion between cells. This study showed a new kind of regulating mechanism. The cancer stem cell sample CD90+ cells can promote angiogenesis and the tumor microenvironment [94]. This research proposed that lncRNA*H19* can also serve as a potential therapeutic target in liver cancer. Other studies showed that exosomal lncRNA *TUC339*, which derives from liver cancer cells, can regulate the tumor microenvironment and can be used to adjust the growth and adhesion ability of tumor cells via the horizontal transformation of exosomes in cells [95].

In recent years, researchers have found that an lncRNA called *lncARSR* (lncRNA Activated in RCC with Sunitinib Resistance) is closely related to sunitinib resistance in clinical treatment [60]. *lncARSR* can be used as a competitive endogenous RNA (ceRNA), when combined with *miR-34/miR-449*, to promote the expression of AXL, c-MET in renal carcinoma cells, and then realize the promotion of sunitinib resistance. However, this bioactive lncARSR is delivered to the recipient cells by exosomes, thereby contributing to the spread of sunitinib drug resistance. Targeted blockade of *lncARSR* or the use of AXL/c-MET inhibitors may aid in treating renal cell carcinoma that is resistant to sunitinib. *lncARSR* may serve as an effective molecular marker and intervention target to improve the therapeutic efficacy of sunitinib in renal cell carcinoma and provide a new approach and perspective for the individualized target therapy of renal cell carcinoma.

Many studies have shown that the expression of long noncoding RNA *ZFAS1* has increased in cancers, and some scholars have linked *ZFAS1* to the exosomes. The expression of long noncoding RNA *ZFAS1* is up-regulated in the serum of patients with gastric cancer, in whom exosomes can promote the proliferation and migration of gastric cancer cells by transmitting the *ZFAS1* [96]. Exosomes can up-regulate cyclin D1 and accelerate the cell cycle. Bcl-2 simultaneously increases time the down-regulation of Bax, which can increase the level of ERK phosphorylation. Exosomal *ZFAS1* can increase the proliferation and invasion of gastric cancer cells. Many studies confirmed that the expression of *ZFAS1* is up-regulated in cancers and proved that it can compete with *miR-150* as ceRNA; further, the expression of *miR-150* can inhibit *ZFAS1* [97]. Other studies have shown that *ZFAS1* is associated with EMT in gastric carcinoma [98]. Whether these biological mechanisms are associated with biological function of exosomes requires further research. The study confirmed that *ZFAS1* may play a role in the tumorigenesis of gastric cancer and can serve as a novel target for treating gastric cancer.

Research found that the lncRNAs in plasma can be protected by exosomes and expressed stably, and researchers have confirmed that the expression of *LINC00152* is significantly increased in the plasma of gastric cancer patients. Its expression level is almost equal in plasma and exosomes [99]. *LINC00152* can be used as a potential and stable molecular marker of gastric cancer. Some studies confirmed that exosomal lncRNAs play important roles in tumor development and progression. *HOTAIR, MALAT1* and *MEG3* can be used as targets of cervical cancer in early diagnosis [100]. The lncRNA *H19* can increase the expression of angiogenic factor VEGF and its receptor VEFG-R1, both of which can be used as potential drug targets in hepatocellular carcinoma [101]. In the development of cancer, many studies have confirmed that a variety of lncRNAs is closely related to the development of cancer. The lncRNAs *HULC*, lncRNA*H19*, *HEIH,* and *MVIH* can promote the growth of cancer cells. The lncRNAs *HOTAIR* and *PVT1* can promote the proliferation of cancer cells. The lncRNAs *MALAT1, LET* and *ATB* can promote the metastasis of cancer cells. Whether these exosomal lncRNAs are closely related to cancers deserves further discussion [62, 100, 101].

### Exosomal circRNAs in tumor metastasis

circRNA is formed by a downstream 5 splice site that is associated with an upstream 3 splice site and is more stable than linear RNA. Its formation can be divided into the phases of intron cyclization, Lasso driving cyclization, intron pairing, driving and cyclization. In the beginning, circRNAs were considered a by-product of splicing errors and did not receive extensive attention. In recent years, with the development of sequencing technology, more circRNAs have been identified [102–105].

Exosomal circRNA is novel and stable. It may serve as a new tumor marker and provide new ideas for the diagnosis and prognosis of tumors. circRNAs may play an important role in cell communication. Some recent studies have confirmed that circRNAs can be used as molecular sponges for miRNAs [106–108], which can competitively inhibit the biological activity of miRNA. *CiRS-7* can be used as a natural molecular sponge for *miRNA-7* to regulate gene expression, regulate transcription and splicing, and regulate parental gene transcription [81, 109]. Researchers speculated that functional exosomal circRNAs participate in intercellular communication. *ciRS-7/CDR1* can serve as a miRNA molecular sponge, which can combine with special miRNA, and can transfer biological information to a specific position to achieve the efficient transmission of biological information and thereby promote the spread of cancer [102].

Studies have shown that the development of colon cancer is closely related to circRNA. The content of exosomal circRNAs is far greater than that found in cells [110]. CircRNAs may regulate the development of colon cancer and serve as molecular markers for cancer.

The expression levels of exosomal circRNAs are stable and abundant. However, the mechanism is unknown and may be attributed to the special protection of exosomes, the features of a specific sequence, or the protection of certain proteins [103]. Exosomal circRNAs are likely to become new markers for cancer therapy and molecular therapeutic targets.

## THE MECHANISM OF EXOSOMAL NON-CODING RNAS INVOLVED IN TUMOR METASTASIS

**Figure 2:**
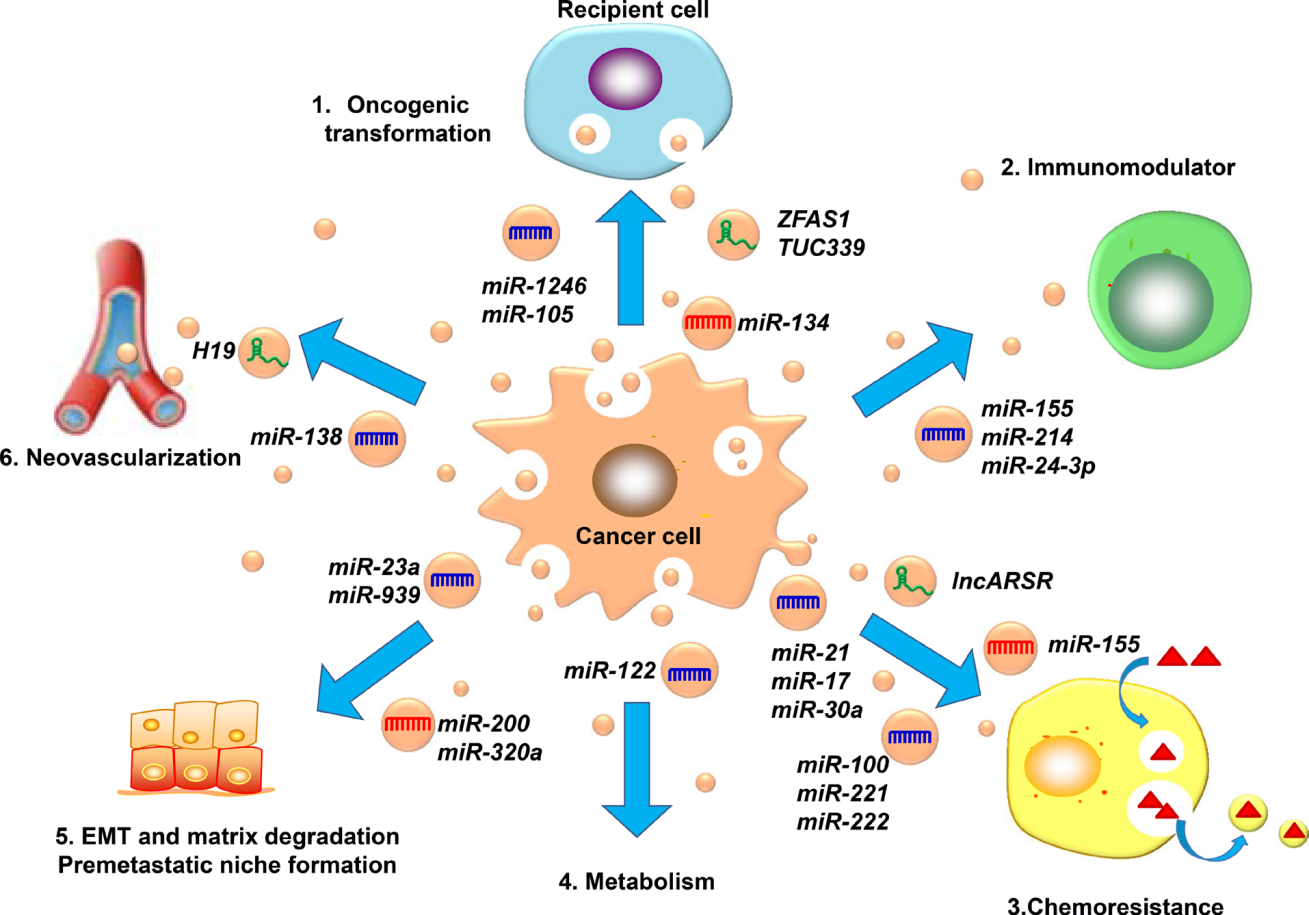
The mechanisms of the exosomal ncRNAs involved in cancer metastasis (**1**). Horizontal transmission of oncogenes, (**2**). Regulate the immune system as immunomodulators, (**3)**. Participate in tumor chemotherapy resistance, (**4**). Regulate tumor metabolism, (**5)**. Influence the progression of EMT and cancer premetastatic niches, (**6**). Promote angiogenesis.

Invasion and metastasis are the basic characteristics of malignant tumors. They are also the pathological bases for tumor recurrence, disease deterioration and, ultimately, death. There are many mechanisms that affect tumor metastasis and are related to a variety of pathways. Exosomal non-coding RNA can also contribute to tumor metastasis through a variety of mechanisms [111]. Figure 2 shows that the pathways of exosomal ncRNAs regulate tumor metastasis.

### Transmission of oncogenes

Exosomal non-coding RNA can affect the proliferation, invasion and migration of tumor cells through specific biological action and can further affect the occurrence and development of tumor metastasis [39]. *miR-1246*, for instance, regulates the invasion and metastasis by regulating DENND2D. *miR-105* destroys vascular endothelial cells by targeting the tight junction protein ZO-1 and promotes metastasis. The lncRNA *ZFAS1* promotes the proliferation and migration of gastric cancer cells via the transmission of the exocrine. *miR-134* can inhibit STAT5B, Hsp90 and Bcl2 and can further inhibit the proliferation, invasion and metastasis of tumor cells. *miR-142-3P* can be selectively isolated from exosomes, thus promoting the development of the malignant phenotype of tumor cells.

### Interfere with the immune system to promote tumor metastasis

Tumor cells can evade the immune response through various mechanisms, such as a lack of histocompatibility complex (MHC)I and the expression of MHC II, mutation and degradation of tumor associated antigen, and blocking immune molecules to increase the tolerance of the immune system. Tumor cells can also inhibit NK cells and raise CTL cells by regulating the output of VEGF, IL-10, TGF-β, and adenosine ROS immunosuppressive molecules, thus damaging the immune response [112], and are not recognized by immune cells to escape immune surveillance [113, 114]. The improvement of tumor cell proliferation and the increased instability can cause the development of different types of antigens. Exosomal CD39 and CD73 exist in cancer cells. These two molecules can generate adenosines, which can restrain immunity; then, tumor cells can regulate immune cells [115]. Exosomes can influence the development of tumor metastasis by affecting the immune function, such as *miR-24-3p* targeting *FGF11* to inhibit T cell function. Macrophages can secrete exosomes that contain abundant *miR-155*, which promotes the expression of TNF-a, IL-6, and IL-23 while increasing the expression of CD40, CD63, CD81, MCH-I, MyD88, and NF-kB. The research has shown that *miR-155* is involved in the inflammatory response [116]. Studies have reported that tumor-derived exosomal *miR-214* inhibited the PTEN protein expression level to induce the secretion of IL-10 by Tregs, leading to the host organism immune escape to promote tumor growth [117].

### Participate in drug resistance to promote tumor metastasis

Multiple drug resistance in tumor cells and tumor metastasis are the two main factors contributing to the failure of cancer treatment. Multidrug resistance (MDR) in tumor cells can cause resistance to drugs that are administer to inhibit cell growth and can cause toxicity. Numerous studies have shown that exosomes blockade can block the response to cell growth therapy [118]. Studies have reported that breast cancer cells can transmit *miR-100, miR-222, miR-30a,* and *miR-17* via exosomes to decrease the drug resistance to doxorubicin and paclitaxel [119]. Exosome can participate in the mechanisms underlying cancer drug resistance to promote tumor metastasis. *miR-21* moves from the stromal cells to the ovarian cancer cells via the transport of exosomes and targets *APAF1* to cause Taxol resistance in ovarian cancer cells [63]. There have also been reports that macrophages can deliver *miR-21* to the stomach cancer cells via exosomes, thereby inhibiting the apoptosis of gastric cancer cells by down-regulating *PTEN* to activate the PIK3/AKT signaling pathway, and can promote cisplatin resistance in gastric cancer cells [120]. The competitive combination of *lncARSR* with *miR-34/miR-449* increases the expression levels of AXL and c-met though exosomes to promote the spread of drug resistance. An increase in *miR-155* in the pancreatic duct adenocarcinoma (PDAC) can induce exosome secretion. By promoting the anti-apoptotic activity produced by chemotherapy drug resistance, exosomal *miR-155* will be delivered to other PDAC cells and can effectively weaken gemcitabine (GEM) resistance [76, 121]. The fibroblasts secrete *miR-221* into tumor cells, which can bind to hormone therapy targets, resulting in tumor hormone therapy resistance (HTR) [122].

### Involvement in tumor metabolism to promote tumor metastasis

Tumor cell metabolism refers to normal cells that are under the action of a carcinogenic factor and develop a continuous proliferation of tumor cells. Similar to increases in glucose intake, lactic acid accumulation or nucleic acid synthesis can reinforce metabolic changes. The three most remarkable features of tumor cells, including immortality, mobility and the loss of contact inhibition, have the most direct relationship with tumor cell metabolism. Molecular biological studies of metastatic tumors have found that the main carcinogenic signaling pathways converge at the end of the tumor cell metabolism. Metabolic changes are essential in the development of tumor cells. Therefore, some research teams have explored the mechanisms of tumor metastasis from the perspective of tumor sugar metabolism; e.g., *miR-122* inhibits glycolysis by downregulating the pyruvate kinase. Recent studies have reported that the pyruvate kinase M2 (PKM2) plays an important role in the release of exosomes [65]. This process is a key step in exosome secretion, and the release of tumor cells is closely related to the general and metabolic transformation of tumor cells.

### Participate in EMT to promote tumor metastasis

EMT is an important process in the early stage of tumor invasion and metastasis. An important marker of EMT is the down-regulation of E-cadherin. Many transcription factors can inhibit the expression of E-cadherin. For example, Snail/Slug family proteins, Twist, ZEB1, SIP1 and the E12/E47, Wnt, TGF-β, and nuclear factor-κB (NF-κB) pathways play an important role in the EMT process. They can promote the occurrence of EMT by affecting the transcription inhibitory factor of EMT [51]. Many studies have shown that a variety of exosomal miRNAs may influence the signaling molecules that are related to the EMT pathway. Exosomal miRNAs can promote or inhibit tumor metastasis by influencing the occurrence of EMT. For example, exosomal *miR-23a* can regulate the development of EMT by influencing TGF-β. Exosomes can induce the transcriptional activation of TCF/LEF and activate the Wnt pathway in lung cancer cells to affect the occurrence and development of tumor metastasis. *miR-939* inhibits VE-cadherin and destroys endothelial barrier function. *miR-200* can directly target E-cadherin’s transcriptional repressors ZEB1 and ZEB2 and can up-regulate the expression of E-cadherin and inhibit the motility of mRNA. *miR-320a-PBX3* can inhibit the activation pathway of MAPK, thereby inhibiting the occurrence of EMT and down-regulating the expression of cyclin dependent kinase 2 (CDK2) and MMP2. Therefore, *miR-320a-PBX3* can inhibit tumor development. The loss of p85α expression in the stroma of breast cancer can cause stromal fibroblasts to acquire the characteristics of cancer associated fibroblasts (CAF). Exosomes carry Wnt10b and induce EMT via the classical Wnt pathway, which can promote cancer progression by modifying the stromal cells and epithelial tissue remodeling of the tumor micro-environment [123].

### Modulate vascular system to promote tumor metastasis

*miR-138*, an exosomal ncRNA that is derived from glioblastoma, enhances tumor angiogenesis and promotes cancer development. The exosomes originating from glioma stem cells promote the angiogenesis of endothelial cells via the *miR-21*/VEGF signaling pathway [124]. The increased expression of angiogenic factor (VEGF) and its receptor VEGF-R1 caused by lncRNA *H19* affects the development of metastatic tumors.

Many researchers have also explored the effects and functions of exosomal ncRNAs through other approaches. For example, some non-coding RNAs in exosomes can play vital biological roles by acting as ceRNA. Further, *lncARSR* combined with *miR-34/miR-449* can increase the expression levels of AXL and c-MET. This promotes the development of metastatic disease by promoting the dispersion of drug resistance. *miR-19a* promotes brain metastasis by inducing the loss of *PTEN* [8]. We need to conduct more in-depth studies to explore new pathways by which exosomal ncRNAs can promote cancer metastasis.

### Summary

In recent years, studies on the mechanism of exosomal non-coding RNAs have increased. However, deeper mechanisms are still to be found in the exosomes. The main challenges in studying exosomes include how they participate in the physiological and pathological processes and dissecting the transfer of exosomes from cell to cell [125, 126]. In this review, we have summarized various exosomal non-coding RNAs that play key roles in tumor metastasis. This review provides a detailed description of the function of exosomal ncRNAs, which will offer new insights and provide novel therapeutic approaches in malignant tumor metastasis.

Exosomes are membranous vesicles that are secreted into the extracellular space by a variety of cells and play important biological functions in intercellular communication. Many sources of exosomal ncRNAs have been shown to be closely related to human malignancies. As the potential functions of exosomes are gradually discovered, it is critical to explore the structures of these vesicles and their social domains. This will give aid in studying the deeper functional mechanisms of exosomes [13, 127, 128]. Currently, the main research approach is to explore the relative miRNAs and protein levels of exosomes [129, 130]. We believe that the lncRNA, circRNA, and other new components will be used in a broader field of exosome investigations [131]. This method of transferring small packages will provide new perspectives for cancer treatment and prognosis and will give more novel ideas to examine tumor metastasis and unique clinical paths.

Exosomal ncRNAs may serve as very important biomarkers for disease diagnosis, prognosis, and treatment. Many studies have reported that there are important cancer-associated RNAs and protein markers in exosomes, but because of their small size and low density, it is not easy to isolate exosomes. Because of the complexity of extraction methods, it is difficult to study the exosomes. The current mainstream exosome isolation techniques include ultracentrifugation-based isolation techniques, ultrafiltration, immunoaffinity capture-based techniques, and exosome precipitation [132, 133]. Recently, a new method for the rapid isolation and identification of exosomes has been found. The core of this technology is an alternating current electrokinetic (ACE) microarray device. Such a device may have more than 1000 electrodes, and its surface is coated with a thin layer of porous water gel. In the dielectrophoretic high field area, the exosome mass is enriched. Exosomes are enriched at the chip electrodes and can be analyzed and identified directly via scanning electron microscopy and immunofluorescence [134]. In addition, the novel microfluidics-based isolation techniques may make it more convenient to explore the exosomal ncRNAs [135, 136]. There are also many emerging technologies for extracting exosomes. The advent of more advanced exosome extraction techniques will greatly accelerate the development of exosomal ncRNA research.

Exosomal ncRNAs play important roles in malignant tumor metastasis. They are hoped to have an indispensable role in targeted drug delivery and clinical therapy. At the same time, the development of more efficient exosome separation techniques will promote exosome research. The anomalous expression levels of exosomal ncRNA may indicate cancer or cancer progression. Exosomal ncRNAs are thus promising biomarkers. Further study of exosomal ncRNAs may provide an effective minimally invasive strategy for the diagnosis, prognosis and treatment of cancer. The discoveries of exosomal ncRNAs have opened a potential market for more exciting clinical applications in the near future.

## Abbreviations

ncRNA: non-coding RNA
LncRNA: Long non-coding RNA
miRNA: micro RNA
ECM: Extracellular matrix
EMT: Epithelial-mesenchymal transition
PDAC: Pancreatic ductal adenocarcinoma
GEM: Gemcitabine
HIF-1 α: Hypoxia-inducible factor-1 α
ZO-1: Tight junction protein
iHEC: immortalized human endothelial cells
MVP: Major vault protein
*HOTAIR*: *HOX* transcript antisense RNA
ceRNA: Competitive endogenousRNA
lncARSR: lncRNA Activated in RCC with Sunitinib Resistance
CAF: Cancer associated fibroblasts
MDR: Multidrug resistance
HTR: Hormone therapy resistance
*PTEN*: Phosphatase and tensin homolog
